# UVB Light as a Source of Vitamin D for Indoor-Housed Gestating Sows

**DOI:** 10.3390/ani15213185

**Published:** 2025-11-01

**Authors:** Sine Stricker Jakobsen, Jette Jakobsen, Sheeva Bhattarai, Jens Peter Nielsen

**Affiliations:** 1 Department of Veterinary and Animal Sciences, University of Copenhagen, 1870 Frederiksberg C, Denmark; sine_s_j@hotmail.com (S.S.J.); sheevabhattarai@gmail.com (S.B.); jpni@sund.ku.dk (J.P.N.); 2 National Food Institute, Technical University of Denmark, 2800 Kongens Lyngby, Denmark

**Keywords:** UVB light, vitamin D, 25-hydroxyvitamin D, gestating sows

## Abstract

Vitamin D has been shown to influence growth in both fetuses and piglets. Pigs housed indoors rely solely on their feed as a source of vitamin D since they are never exposed to natural sunlight. UVB light is effective in improving the vitamin D status in sows and piglets housed indoors. This study aimed to investigate an innovative method for UVB lighting to enhance the vitamin D status of gestating sows to a level that could positively impact the growth of both fetuses and suckling piglets. A total of 386 sows were included at the time of insemination. Of these, 197 were exposed to a daily dose of UVB light, and 189 sows served as a control group. Both sows and piglets in the UVB group had significantly higher vitamin D levels in serum samples at the time of farrowing than sows and piglets from the control group. No statistically significant differences in litter weight or the number of liveborn or stillborn piglets were observed between groups. UVB light installed in an electronic sow feeder was successful, but, unfortunately, the lamp used in the study was unable to induce the production of vitamin D to the desired level in the sows. Further studies are needed to investigate the effect of higher doses of UVB light.

## Simple Summary

A randomized parallel study on UVB light exposure was conducted in a Danish herd. The aim was to improve the vitamin D status in gestating sows and their offspring by exposure to UVB light during visits to electronic sow feeders. A total of 386 sows were included in the study and divided into a group of 197 sows receiving a daily dose of UVB light and a control group of 189 sows under standard indoor production conditions. Both groups received standard vitamin D supplementation in their feed. Serum levels of vitamin D were significantly higher for UVB-exposed sows and their offspring compared to the control group. However, the vitamin D levels in UVB-exposed sows were lower than those previously reported in studies of outdoor-housed sows. No statistically significant difference in litter weight at birth, number of liveborn piglets, or number of stillborn piglets was observed between groups. The study showed that the vitamin D status of gestating sows was improved through UVB exposure using lamps installed in electronic sow feeders. Further studies using more efficient lamps are needed to investigate whether a further increase in levels of UVB-derived vitamin D can improve the birth weight of piglets and litter size in sows.

## 1. Introduction

Vitamin D is a fat-soluble vitamin that plays a role in numerous functions in the body, including body growth and the immune system [1,2]. Beyond these well-known postnatal functions, emerging research indicates that vitamin D is vital for establishing and maintaining pregnancy, as well as regulating fetal growth in various mammalian species [3,4]. For pigs, a tendency for fewer stillborn [5], and for humans, increased birth weight [6] was associated with vitamin D intake. Vitamin D can be obtained by pigs and most mammals either through their diet or via UVB exposure of the skin. The provitamin D_3_, 7-dehydrocholesterol, present in the skin, is converted to previtamin D_3_ by exposure to UV light below 315 nm_._ Subsequently, previtamin D_3_ is isomerized to cholecalciferol (vitamin D_3_) at body temperature. Vitamin D_3_ is a seco-steroid, which, in the liver, is hydroxylated to 25-hydroxyvitamin D_3_ (25-OHD_3_). A similar process occurs with provitamin D_2_ (ergosterol), which is derived from fungi and subsequently converted in the liver to 25-hydroxyvitamin D_2_ (25-OHD_2_). In mammals, the accepted biomarker for assessing vitamin D status is the serum level of 25-OHD_3_ and 25-OHD_2_. Both 25OHD_3_ and 25-OHD_2_ are further metabolized in the kidneys to form 1,25 hydroxyvitamin D_3_ and 1,25 hydroxyvitamin D_2_, respectively—the biologically active forms of vitamin D.

Exposure of the skin to UVB will, besides the production of vitamin D, lead to the production of other vitamin D compounds, which lack biological activity. Due to a self-regulating mechanism, excess production of vitamin D in the skin will not lead to toxicological levels [7]. Nevertheless, prolonged UVB exposure can cause DNA damage, which in humans increases the risk of developing skin cancer.

Although UVB light in the range 290–315 nm is present in sunlight, the effect on vitamin D production depends on the season, latitude, and area of the skin exposed [8]. Studies in wild boar showed a seasonal variation that is similar to the variation observed in humans [9,10]. In a Danish setting, the vitamin D status for sows during late summer was 67 ng 25-OHD/mL serum [11].

Modern pig production is based on indoor herds with high biosecurity standards that do not allow the pigs to be exposed to outdoor conditions. This means that pigs rely on vitamin D in their diet. In Europe, feed supplemented with vitamin D is regulated by the European Union [12]. Feeding vitamin D_3_ at the maximum allowed level (2000 IU vitamin D_3_/kg feed) resulted in 26 ng 25OHD_3_/mL serum [5].

Using UVB light as a source of vitamin D for pigs housed indoors could potentially improve their vitamin D status and thereby improve health and production parameters in modern facilities.

Our previous studies showed that it is possible to improve the vitamin D status of sows housed indoors in modern facilities by exposure to UVB light [13]. However, there is a need to evaluate more convenient methods of exposing pregnant sows to UVB light. The aim of this study was to test an innovative method for UVB exposure of gestating sows during feeding in automated feed stations and thereby increase the vitamin D status and birth weight and reduce the number of stillborn piglets.

## 2. Materials and Methods

### 2.1. Design and Herd

A randomized parallel study was carried out in Denmark in a commercial herd with 1800 sows. The majority of sows were hybrid crosses between Danish Landrace and Yorkshire (Danbred), either LY or YL, or backcross hybrids LYL or YLY, and a small number (of the sows—14 in total—were purebred Yorkshire (YY). The sample size calculation was based on a significance level of 0.05, power of 80%, standard deviation of litter weight of 4544 g, which was observed in an unpublished study performed in the same herd, and a minimum detectable difference in litter weight of 1300 g between the two studied groups. This resulted in a group size of 193 sows. The study was approved by the Danish Animal Experiments Inspectorate (Approval number 2020-15-0201-00678) and was carried out from January 2021 to July 2021.

The farm was a commercial herd located in Denmark with a health declaration of “SPF + Myc”, which meant that the farm was infected with *Mycoplasma hyopneumoniae* but declared free of the following diseases: porcine pleuropneumonia, PRRS, swine dysentery, atrophic rhinitis, mange, and lice [14]. Sows were loose housed during gestation and were fed liquid feed in electronic sow feeders (Big Dutchman International GmbH, Vechta, Germany). After insemination, each weekly batch of sows and gilts was housed in a control section, with gilts in one pen and sows of parity > 1 in another. After a confirmed pregnancy in week 4, the sows and gilts from one weekly batch were moved to a pen in the gestation unit, where they would remain for 11 weeks. During gestation, the sows had access to straw and were kept in stable groups with up to 80 sows/gilts per group/sow feeder from 4 weeks after insemination until 1 week prior to farrowing, when they were moved to the farrowing unit. The electronic sow feeders would register the sow ID number by reading the electronic ear tags. Feeding began at 9.30 pm and ended the next day at 3.30 pm. During this period, the sows were able to enter the feeding station one at a time and eat their daily ration of feed.

To achieve standard supplementation with vitamin D, the addition of vitamin D to the feed was changed from 50 µg 25-OHD_3_(Hy•D, DSM-Firmenich, Kaiseraugst, Switzerland)/kg feed to 20 µg vitamin D_3_/kg feed 6 weeks before the first weekly batch was inseminated. The addition of 20 µg vitamin D_3_/kg feed is the recommended level according to the Danish Pig Research Centre [15].

A total of 493 sows (parity 1–6 and inseminated with Duroc semen) from seven consecutive weekly batches were included in the trial within 1 week of insemination. Sows were allocated to one of two groups by systematic random selection. All sow numbers from one weekly farrowing batch were listed in Excel next to a list of consecutive randomly generated numbers. After sorting the randomly generated numbers, the first half was allocated to the UVB group and the second half to the control group.

The UV group totaled 196 animals and consisted of 32 gilts and 164 sows (parity > 1). The control group consisted of 189 animals here, of which 27 were gilts and 162 were sows. From each of the 7 weekly batches, ten gestating sows (five from the control group and five from the UVB group) were selected for blood sampling during the week of insemination. In total, 6 gilts and 64 sows were blood sampled.

Gestating sows were monitored daily by farm staff, and animals with health concerns were either treated, observed closely, or moved to a sick pen according to the severity of the case. Sows/gilts were visually inspected, and the daily feed output from the electronic sow feeders was monitored. In the farrowing section, sows were visually inspected daily, and feed intake per day was monitored. Obstetric aid was provided by trained farm staff when necessary [16,17].

### 2.2. Recordings and Samplings

No piglets were moved within the first 24 h after completion of farrowing, as recordings and initial blood samples were performed during this time. The number of liveborn and fully developed stillborn piglets was recorded, as well as the number of liveborn piglets that died during the time between the farrowing and sampling. Due to practical limitations, the number of mummies was not recorded. Shortly after blood sampling and weighing, farm staff adjusted litter size by moving the largest and/or smallest piglets to foster sows. Litter size varied between sows and was individually determined based on the number of functional teats, past maternal performance, and sow health status. Piglets within the study were only moved to other sows from the same treatment group. Excess piglets that could not be placed with a sow from the same treatment group as their mother sow had their ear tag removed and were excluded from the trial. Such piglets were registered as “moved to sow not in experiment”.

Piglets that died during the suckling period were registered using their ear tag number, body weight, and date of death.

Antibiotic treatment was administered by farm staff as part of their normal work routine. For sows in the gestation unit, five different treatment causes were used: pain, mastitis, agalactia, metritis, and lameness, as well as the common combination of mastitis/metritis/agalactia (MMA). Treatments were registered individually for each sow according to Danish legislation [16,17].

**Figure 1:**
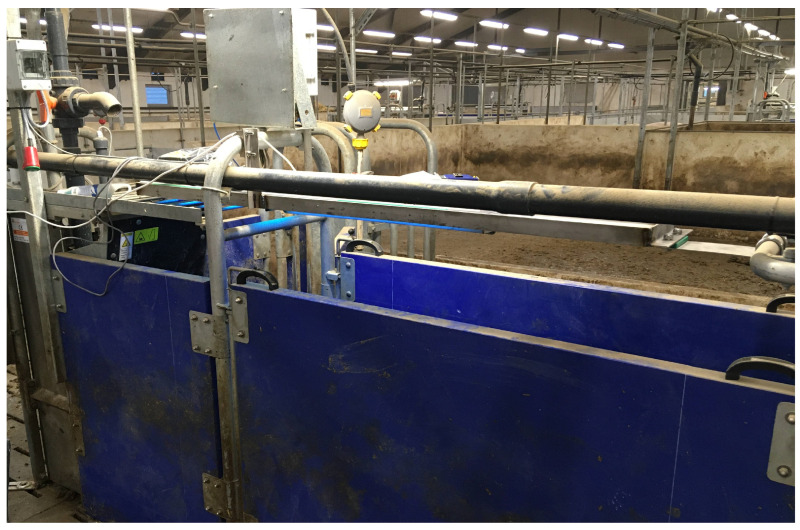
Electronic sow feeder with UVB lamp turned on. The photo was taken during preparations for the study. To avoid unintended exposure of control sows, a UVB-proof plastic top covered the feeding station throughout the study.

Each sow in the UVB group was exposed to 3 min and 15 s of UVB light daily during gestation while they were fed in the electronic sow feeder (Figure 1). The exposure time was determined by the duration of eating and was adjusted to ensure that even the fastest-eating sow, which took 4 min and 1 s, would still be in the feeding station at the end of the exposure period.

Lamps were connected to a timer that was also connected to the feeding system. When sows from the UVB group entered the feeding station, they triggered the timer so that the lamp would come on for 3 min and 15 s. The system would only trigger the timer once per day per sow. Lamps were attached at a height of 123 cm above the floor, which was equivalent to a mean distance of 35 cm from lamp to sow. Lamps consisted of two identical 120 cm UVB fluorescent light tubes (UVB-313 40 W, Q-Lab Corporation, Budapest, Hungary). The lamps were placed in a fixture and covered by UVB-penetrable plexiglass to protect the light tubes from the environment. The spectral irradiance of the light tubes was measured at the Technical University of Denmark, Department of Electrical and Photonics Engineering [18]. The measurements were used to calculate the Standard Erythemal Dose (SED) and the ability to produce vitamin D (Vitamin D dose) [8,19]. For SED, the irradiance at each wavelength (W/m^2^/nm) was multiplied by the redness factor and by the exposure time in seconds and divided by 100 (as 1 SED is defined as 100 J/m^2^) [19]. To calculate the vitamin D dose (vitD_3_ J/m^2^), the irradiance at each wavelength (W/m^2^/nm) was multiplied by the corresponding factor from the action spectra for vitamin D synthesis [8] and by the exposure time in seconds. The UVB exposure was calculated to provide each sow with a daily dose of 1.4 SED and 164 vitD_3_ J/m^2^.

Throughout the study, the lamps were cleaned weekly using a portable compressor to remove dust. Following each cleaning, UV irradiance was measured using a handheld UVB photometer (ILT1400-BL, International Light Technologies, Peabody, MA, USA) to ensure that both the UVB irradiance and, consequently, the UVB dose remained consistent during the study period.

Systematic random sampling was used to select the 70 sows for blood sampling. Five sows from the control group and five sows from the UVB group were selected from each weekly batch and blood sampled in weeks 1, 8, and 16. In total, 35 sows from each group were sampled in week 1 of gestation. Of these, 13 sows did not become pregnant and were excluded before the second sampling. An additional two sows were excluded before the third sampling due to health issues and were moved to the sick pen. This resulted in 27 sows from the UVB group and 28 sows from the control group having blood samples taken at all three sampling points. The sampled sows were parity 1–5, with 6 being parity 1, 19 being parity 2, 15 being parity 3, 12 being parity 4, and 3 being parity 5. One piglet from each of the blood-sampled sows was selected by systematic random sampling for the collection of blood. Additional piglets were selected by first selecting the litter and then selecting the piglet, both using systematic random sampling. In total, 144 piglets were sampled at birth, with 74 from the UVB group and 70 from the control group. Piglets of each sex were evenly represented in the treatment groups, and in total 77 piglets were female and 67 were male. The mean birthweight of piglets in the UVB group was 1338 ± 357 g and 1327 ± 372 g for control piglets.

Fifteen piglets from the UVB group and 16 piglets from the control group died during the lactation period; thus, 59 piglets from the UVB group and 54 from the control group were sampled again at weaning.

Blood samples from sows/gilts were obtained from the jugular vein using a 18 G × 1.5” needle (BD ref. 360748, Becton Dickinson, Franklin Lakes, NJ, USA), a vacutainer holder, and 4 mL or 10 mL clot activator tubes (BD ref 369032 or 367896). Piglets were blood sampled using a 22 Gx1” needle (BD ref. 360210), a vacutainer holder, and a 4 mL blood activator tube (BD ref. 369032). Blood samples were transferred to a cooling at 5 °C within 2 h of collection and kept for up to 36 h before centrifugation at 2500× *g* for 15 min. The resulting serum was transferred to separate vials and stored at −80 °C for subsequent analysis.

Serum samples were analyzed following a method previously described by Barnkob et al. [20]. From each serum sample, 100 µL was mixed with acetonitrile to precipitate the protein. Samples were further cleaned up by solid-phase extraction and derivatized by 4-phenyl-1,2,4-triazoline-3,5-dione. Liquid chromatography coupled with tandem mass spectrometry was used to separate and quantify 25-OHD_3_ and vitamin D_3_.

Medical treatments were registered according to Danish legislation [13,14] and added to the dataset.

### 2.3. Statistics

Statistical analysis was performed using the statistical software RStudio (version R.4.4.0, Rstudio Inc., Boston, MA, USA). Mean blood levels of vitamin D_3_ and 25-OHD_3_ from the first sow sampling were analyzed using a student’s *t*-test to ensure that there were no significant differences between groups before UVB treatment was initiated. The level of significance for all analyses was set to *p* < 0.05.

Data were accessed for normality, and if possible, data were transformed to obtain normal distributions. However, variables that could not be described by a normal distribution were reassessed, and appropriate alternative distributions were used in the analysis.

The 25-OHD_3_ results from the second and third sampling were transformed by Tukey’s ladder of power transformation to obtain a normal distribution. The 25-OHD_3_ results in sows were analyzed separately for the second and third sampling using a linear model with breed, group, and parity group (with gilts in one group and sows in another) as fixed effects. Vitamin D_3_ results were log-transformed to obtain a normal distribution. Results were analyzed using a linear model with breed, group, and parity as fixed effects.

The total number of piglets born was analyzed using a generalized linear model with a Poisson distribution. The Poisson distribution was used because of the independent and discrete nature of the data. In the model, group, treatment, weekly batch, and parity group were fixed effects. Similarly, the number of liveborn piglets was analyzed using a generalized linear model with a Poisson distribution. The number of stillborn piglets per litter was analyzed using a generalized linear model with a negative binomial distribution. Group, parity group, weekly batch, and liveborn piglets were fixed effects.

Litter weight, defined as the total weight of live and stillborn piglets, was transformed by Tukey’s ladder of transformation to obtain normal distribution and analyzed using a linear model with parity group, weekly batch, and total number of piglets born as fixed effects. The total weight of liveborn piglets per litter (litter weight) was transformed by Tukey’s ladder of transformation to obtain a normal distribution and then analyzed using a linear model with group, parity group, weekly batch, and liveborn piglets as fixed effects.

The 25-OHD_3_ results in piglets were analyzed using a linear mixed effects model with group, sex, gestation days, birth weight, treatment of sow, and litter weight as fixed effects and sow as a random effect. The individual birth weight of piglets was checked for normality using a histogram plot. Data were then analyzed using a linear mixed effects model with group, total number of piglets born, litter weight, parity, gestation days, and weekly batch as fixed effects and sow as a random effect. Weaning weight was analyzed using a linear mixed effects model with group, total number of piglets born per litter, litter weight, birth weight, parity, whether the piglet was removed from the dam, sex, and treatment as fixed effects and sow as a random effect. Average daily gain was analyzed using a linear mixed effects model with group, litter weight, birth weight, whether the piglet was removed from the dam, parity, liveborn piglets, treatment of sow (yes/no), and age at weaning as fixed effects and sow as a random effect.

## 3. Results

### 3.1. Sows

**Figure 2:**
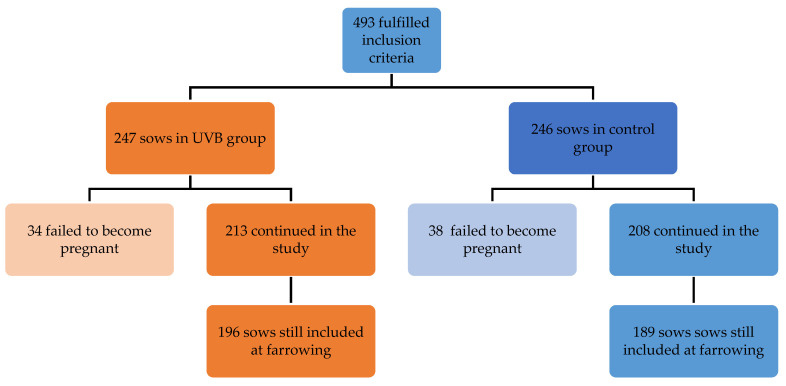
Group distribution of 493 sows and gilts included in the first week after mating. Only sows that became pregnant and farrowed were included in the final study groups.

In total, 385 sows finalized the study. The selection process for sows/gilts and the distribution into the control group and UVB group are presented in Figure 2. Sow and litter performance results are presented in Table 1.

#### 3.1.1. 25-OHD_3_ and Vitamin D_3_

In week 1 of gestation, no statistically significant differences were observed in the blood levels of 25-OHD_3_ and vitamin D_3_ between the UVB group and the control group. However, a statistically significant difference between groups in 25-OHD_3_ and vitamin D_3_ levels was found in weeks 8 and 16 of gestation (*p* < 0.0001) (Table 1).

Within the UVB group, a statistically significant increase (*p* < 0.0001) in 25-OHD_3_ was observed between weeks 1–8 and 1–16. For vitamin D_3_ in the UVB group, all samples were significantly different from one another (*p* < 0.03). For the control group, a statistically significant difference between weeks 1–8, 1–16, and 8–16 was found for 25-OHD_3_ (*p* < 0.02). For vitamin D_3_ in the control group, the sample from week 8 was significantly different (*p* < 0.003) from samples from week 1 and week 16.

#### 3.1.2. Litter Results

Total born, number of liveborn piglets, and number of stillborn piglets did not differ significantly between treatment groups (Table 1).

### 3.2. Piglets

The sows included in the study gave birth to 7767 piglets between 5 May 2021 and 24 June 2021. Of these, 796 were stillborn and 232 died before they were weighed within the first 24 h after birth. In the suckling period, 760 piglets were registered as dead, 261 were missing, 594 were moved to a sow that was not included in the experiment and were thus excluded, and five piglets were registered twice and were excluded. In total, 5119 piglets were weaned. The preweaning mortality after the first 24 h was 14.8% (477 of 3230 liveborn) and 14.7% (515 of 3509 liveborn) in the UVB and control group, respectively.

The piglet 25-OHD_3_ levels were measured at birth and at weaning. A significant difference between groups was observed for both birth samples and weaning samples (*p* < 0.005 (Table 2). Only five piglets from the control group and 13 piglets from the UVB group had vitamin D levels above LOQ at weaning.

The birth weight of individual piglets was significantly associated with group, total number of piglets born in the litter, litter weight, and gestation days when analyzed in the linear model. Piglets in the control group were significantly heavier at birth compared to the piglets in the UVB group (*p* = 0.04).

Weaning weight and average daily gain were significantly affected by birth weight, parity, antibiotic treatment of the sow, and whether the piglet had been removed from the dam (*p* < 0.02) when analyzed in the linear model. No significant difference in weaning weight was observed between groups. Results for piglets are presented in Table 2.

## 4. Discussion

We have previously demonstrated that UVB light lamps can be successfully installed traditionally above farrowing sows and piglets [19]. Furthermore, for gestating sows, we successfully installed UVB lamps in electronic sow feeders under normal production conditions in a commercial herd, ensuring that the sows automatically received a dose of UVB light while eating (unpublished). The challenge in our first attempt using sow feeders was that the UVB lamp could not withstand the physical force exerted by the sows. In this study, both the physical and electrical constructions functioned well, and none of the sows suffered from erythema caused by the daily UVB dose.

The UVB exposure resulted in a vitamin D response in sows from the UVB group, leading to an increase in their 25-OHD_3_ serum levels compared to the initial values. In contrast, the 25-OHD_3_ serum levels in the control group decreased during the study. The absence of a steady-state vitamin D status in the control group at the start of the study may have reduced the possibility of demonstrating an effect of UV exposure on litter performance.

The decrease in the control group from 24.0 ng 25-OHD_3_/mL serum to 16.5 ng 25-OHD_3_/mL serum was anticipated since a replacement of 50 µg 25-OHD_3_/kg feed with 20 µg vitamin D_3_/kg feed was made six weeks ahead of the trial. A statistically significant difference in 25-OHD_3_ serum levels between the UVB group (30.8 ng/mL) and the control group (16.5 ng/mL) was observed after 11 weeks of UVB exposure. However, the 25-OHD_3_ serum levels after 11 weeks were not as high as we had anticipated. Previous results from exposure of sows to UVB light for 24 days resulted in 25-OHD_3_ levels of 66.9 ng/mL [13]. Currently, there is no consensus on optimal vitamin D levels for sows or other groups of pigs. However, a study conducted on outdoor sows in Denmark during the summer reported serum 25-OHD_3_ levels averaging 67 ng/mL [11]. Another study, including indoor and outdoor housed sows in the United States, Iowa, found levels of 36 ng 25-OHD/mL and 57 ng/25-OHD/mL, respectively [21]. Based on these studies, the optimal vitamin D levels in sows are considerably higher than those obtained in the present studies.

The redness factor measured in this study at 1.4 SED was higher than 1.0 SED used in our previous study, and likewise, the daily vitamin D dose at 164 vit D_3_ J/m^2^ in this study was higher compared to 140 vit D_3_ J/m^2^ in our previous study [13].

The SED indicates the risk for redness, while the vitamin D dose (vitD_3_ J/m_2_) indicates the efficiency of the lamps to enable production of vitamin D_3_ in the skin of the sows. The calculated SED and vitamin D dose were based on the irradiance measured at the very center of the two UVB tubes. This is where the irradiance is highest; the irradiance is lower at more distant parts of the lamps. This means that only a very small area of the sow would be exposed to the estimated dosage. Furthermore, the distance of the lamp to the exposed subject is of importance, as a longer distance will enable a longer exposure time to achieve the set dose, but it might also cause the UVB light to spread out a little more, resulting in a larger exposed area than if the lamp were placed at a shorter distance to the subject. In our previous study of lactating sows, the lamps were placed at approximately 185 cm above the floor [13]. Sows would be either standing or lying down during the exposure of 6 h. In this study, the lamp was placed 126 cm above the floor, and the sows would be standing up during the very short exposure time of 3 min and 15 s. The area of skin exposed to full irradiance in this study is therefore likely to be smaller than the area in our previous study. For humans, it is reported that the amount of vitamin D produced depends on the area of UVB exposed, which could explain the lower-than-expected vitamin D status observed in the sows in our study [22].

A significant difference in the 25-OHD_3_ levels was observed between piglet groups, although concentrations remained very low in both the control and the UVB-exposed group. The levels of 25-OHD_3_ in serum from newborn piglets have previously been reported at similarly low levels on average at approximately 1 ng/mL [23]. Others reported an average serum 25-OHD_3_ concentration of approximately 4 ng/mL; however, this estimate was based only on the 25% of the piglets whose values exceeded the quantification limit of the analytical method [5].

No difference in litter weight or number of stillborn piglets was observed between groups. The UVB group had, on average, 0.7 more piglets per litter than the control group, but this was not a statistically significant difference (*p* = 0.12, Table 1). The higher number of piglets born in the UVB group is probably the reason why these pigs were found to be significantly smaller than the pigs in the control group at birth.

This study demonstrates that vitamin D status in gestating sows can be improved through UVB exposure using lamps installed in electronic sow feeders. This setup worked under practical conditions in a commercial herd without interfering with the daily routines. However, to achieve greater improvements in vitamin D status, more efficient UVB lamps are needed. The setup for this study included a farm with liquid feed in the gestation unit. This meant that the effective eating time was very short, and this was a limitation to the study since we could not provide a vitamin D dose high enough to improve vitamin D levels, as seen in previous studies for sows housed outdoors and indoors with UVB light exposure [11,13].

In an earlier study of sows and piglets in farrowing crates, we employed a strategy involving UVB exposure for six hours per day, divided into two intervals of four and two hours. However, implementing a similar approach in a gestating facility with loose sows moving around is not possible. Farrowing sows were housed in confined spaces, allowing for controlled exposure, whereas pregnant sows had access to larger areas, making consistent UVB exposure more difficult. That said, we adapted our strategy accordingly to ensure controlled exposure in both housing conditions. In the present study, we had one very short exposure period per day. It could be speculated that a long exposure time with lower irradiance is better than a short exposure time with high irradiance, or that dividing the exposure time over more than one time slot would be beneficial in terms of vitamin D production.

As recently reviewed [24], “A multitude of studies indicate that vitamin D has an important role in the prenatal development, immune system, and reproduction of pigs”. Furthermore, optimal vitamin D status has not been assessed, and we still have to understand how vitamin D is involved in the regulation of the immune and reproductive systems. Furthermore, the vitamin D status achieved by free-range pigs could be the indicator for optimal vitamin D status. Also, by UVB-exposure to indoor raised sows we have previously shown that a similar level of vitamin D status can be obtained [13]. When using oral or injectable supplementation, it is possible for sows to receive excess amounts of the vitamin and, over time, develop hypervitaminosis D in response [25]. However, provision of UVB light as the source of supplementary vitamin D production in gestation units would have the advantage of eliminating the risk of hypervitaminosis D due to the self-regulating mechanism involved [7]. In this study, we used a commercial fluorescent lamp. Originally, we aimed to use a UVB LED lamp, but unfortunately, the efficacy was not sufficient for an exposure of 3 min and 15 s. We successfully managed to produce an LED-based UVB lamp, which was characterized by using the same methodology as the lamps in this study, but without delivering a sufficient vitamin D dose to achieve the desired physiological effect. The market for UVB LED has developed, and new studies can potentially take advantage of our finding that UVB-exposure can be part of a feeding station.

When designing the UVB LED lamp, it is essential to consider the specific emission spectrum of the lamp. Vitamin D synthesis peaks at a wavelength of 298 nm; however, it is important to note that the Standard Erythema Dose (SED) doubles as the wavelength decreases from 298 nm to 295 nm, while vitamin D production decreases by only about 2% [8,19]. An optimal ratio between vitamin D production and erythema risk is suggested at 307–308 nm, which should be a target value for future UVB LED lamp design.

Furthermore, it is worth noting that the UVB dose received by the sows depends on several factors, including the type of lamp used and measurement of irradiance, the distance between the lamp and the sow, the surface area of the sow exposed to the light, and the duration of exposure. Also, the construction must be able to withstand the physical force exerted by the sows.

## 5. Conclusions

This study aimed to improve the vitamin D status by exposure of sows to UVB light during feeding in electronic feeder stalls. UVB-exposed sows and their piglets had significantly higher vitamin D levels in serum samples at the time of farrowing than sows and piglets that were not UVB-exposed. No statistically significant differences in litter weight or the number of liveborn or stillborn piglets were observed between groups. The study demonstrated that UVB light installed in electronic feeders was practically applicable. However, since the desired level of vitamin D production in the sows was not reached and further studies are needed to investigate the effects of higher doses of UVB preferably by LED lamps.

## Figures and Tables

**Table 1:** Serum concentration of 25-OHD_3_ and vitamin D_3_ at gestational weeks 1, 8, and 16 in sows from the UVB and control groups, along with farrowing outcomes for both groups.

Sows		UVB Group					Control Group					*p*-Value
		n__sows_^1^	mean	SEM	min.	max.	n__sows_^1^	mean	SEM	min.	max.	
25-OHD_3_ (ng/mL)	Week 1	27	22.8	0.9	16.5	35.0	28	24.0	0.7	17.6	31.3	0.27
	Week 8	27	33.3	1.4	20.1	60.2	28	18.7	0.6	14.3	28.1	<0.0001
	Week 16	27	30.8	1.1	21.0	47.1	28	16.5	0.5	12.5	21.5	<0.0001
Vitamin D_3_ (ng/mL)	Week 1	27	3.0	0.2	1.0	4.8	28	2.7	0.1	1.8	4.4	0.14
	Week 8	27	10.2	0.5	5.4	16.8	28	2.1	0.1	1.1	3.0	<0.0001
	Week 16	27	8.1	0.4	5.2	12.3	28	2.5	0.1	1.4	3.9	<0.0001
**Litters**		**n__litters_^2^**	**mean**	**SEM**	**min.**	**max.**	**n__litters_^2^**	**mean**	**SEM**	**min.**	**max.**	
No. of total born		196	20.5	0.8	7	32	189	19.8	0.8	5	30	0.12
No. of liveborn		196	18.4	0.8	2	26	189	17.7	0.8	4	28	0.13
No. of stillborn		196	2.2	0.4	0	12	189	2.1	0.4	0	16	0.21
Total litter weight (g) ^3^		196	25,951	995	11,605	36,300	189	25,511	914	8395	41,020	0.40
Litter weight of liveborn (g) ^4^		196	23,697	1003	2065	35,560	189	23,222	879	8395	39,710	0.18

^1^ n__sows_ is the number of sows analysed for serum 25-OHD_3_ and vitamin D_3_. ^2^ n__litters_ is the number of litters born by sows in the two groups. ^3^ Total litter weight was defined as the total weight of liveborn and stillborn piglets within a litter. ^4^ Litter weight of liveborn was defined as the weight of all liveborn piglets within a litter.

**Table 2:** Serum concentration of 25-OHD_3_ at birth and weaning in piglets born to sows from the UVB and control groups, along with individual piglet weights at birth and weaning.

		UVB Group					Control Group					*p*-Value
Piglets		n__sampled_^1^	Mean	SEM	min.	max.	n__sampled_^1^	mean	SEM	min.	max.	
25-OHD_3_ (ng/mL)	Birth	74	1.60	0.06	0.5	3.3	70	0.88	0.05	0.5	1.9	<0.000
	Weaning	59	5.6	0.3	2.3	12.4	54	4.4	0.3	0.5	14.1	0.005
**Piglets**		**N ^2^**	**Mean**	**SEM**	**min.**	**max.**	**N ^2^**	**mean**	**SEM**	**min.**	**max.**	
Birth weight, all piglets (g)		4043	1265	5.62	265	2425	3723	1286	6.09	260	2660	0.052
Birth weight, liveborn (g)		3509	1306	5.65	425	2425	3230	1323	6.19	435	2660	0.156
Birth weight, stillborn (g)		416	1039	1.34	265	2110	380	1093	20.62	260	2230	0.07
Weaning weight (g)		2654	6081	35.37	1160	11,780	2465	6053	38.45	1140	13,010	0.597
Average daily gain (g)		2651	195.7	1.35	7.6	432.5	2463	194.1	1.47	13.1	436.3	0.35

^1^ n__sampled_ is a subsample of piglets born to the sows from the UVB and control groups, which was analyzed for serum 25-OHD_3_, and vitamin D_3_ concentration (Table 1). ^2^ N represents the number of piglets recorded in each of the five listed categories.

## Data Availability

The data presented in this study are available on request from the corresponding author. The data are not publicly available due to privacy restrictions.
